# Tear Dynamics During Fenestrated Scleral Lens Wear: A Pilot Study

**DOI:** 10.1007/s44402-026-00102-7

**Published:** 2026-05-06

**Authors:** Damien Fisher, Asif Iqbal, David Alonso-Caneiro, Michael J. Collins, Stephen J. Vincent

**Affiliations:** 1 Contact Lens and Visual Optics Laboratory, Optometry and Vision Science, Centre for Vision and Eye Research, Queensland University of Technology, Brisbane, Queensland Australia; 2 School of Science, Technology and Engineering, University of the Sunshine Coast, Petrie, Queensland Australia

**Keywords:** Corneal oedema, Fenestration, Scheimpflug imaging, Scleral lens, Tear exchange

## Abstract

**Purpose:**

To develop a Scheimpflug-based image analysis technique to quantify tear exchange during scleral lens wear and investigate fluid reservoir tear dynamics during fenestrated lens wear.

**Methods:**

Nine healthy participants wore a scleral lens (KATT™, Capricornia Contact Lenses) with a single 0.3 mm diameter limbal fenestration in one eye for 90 min. Central (0–2.5 mm from the corneal apex) and peripheral (−1.0 to 0 mm from the scleral spur) stromal cornea oedema was measured using optical coherence tomography. Scheimpflug images were obtained during lens wear at multiple time points following the application of sodium fluorescein to the bulbar conjunctiva. These images were exported and annotated manually to select the region of interest (the fluid reservoir) from which the intensity of each pixel was extracted to provide a measure of fluorescent intensity (in arbitrary units [AU] on a scale of 0–255) throughout lens wear across the central 10 mm.

**Results:**

The coefficient of repeatability for central fluid reservoir intensity measurements was 7 AU (on a scale of 0–255 AU). Fluid reservoir fluorescent intensity varied with measurement location (*p* < 0.001), being greater towards the periphery (4 and 5 mm from the centre). On average, intensity differences between the peripheral and central fluid reservoir diminished within 10 min of sodium fluorescein application. Two patterns of tear dynamics were observed and were classified as low and high flow. Low flow participants (*n* = 6) exhibited greater central (3.72× more) and peripheral (2.25× more) corneal oedema, but the difference was not statistically significant.

**Conclusions:**

The ingress and mixing of sodium fluorescein within the fluid reservoir stabilised between central and peripheral locations after 10 min of fenestrated scleral lens wear. Two patterns of tear dynamics were observed (low and high flow), with low flow participants exhibiting greater corneal oedema. Future research utilising the developed technique may provide further insights into tear exchange during scleral lens wear with different fenestration sizes and configurations.

Key Points

- Scheimpflug imaging was utilised to quantify fluid reservoir tear dynamics during fenestrated scleral lens wear across the central 10 mm.
- Tear mixing within the fluid reservoir stabilised between the central and peripheral regions after 10 min.
- Peripheral scleral lens modifications may alter tear dynamics and potentially reduce corneal oedema.

## Introduction

Tear exchange influences oxygen delivery to the cornea during contact lens wear and facilitates the removal of metabolic waste products [1, 2]. Since scleral lenses settle back into the underlying conjunctival tissue throughout the day [3–6], there is minimal tear exchange following the initial settling period, unless there is some lens movement, misalignment of the landing zone with the underlying conjunctival tissue or a lens modification to alter the flow of tears into the fluid reservoir (e.g., a fenestration or back surface channel).

Several studies have examined tear exchange during scleral lens wear by applying sodium fluorescein to the conjunctiva and observing its ingress into the fluid reservoir (the ‘out-in’ method) [7, 8], or by applying the dye within the scleral bowl and measuring its dilution or egress over time (the ‘in-out’ method) [9–11]. An early study [9] used fluorophotometry to examine tear exchange during fenestrated (four eyes) and channelled scleral lens wear (10 eyes) over a period of 1 h and noted a high degree of individual variation. The decay of sodium fluorescein from the fluid reservoir was expressed as an exponential coefficient that reflected the ratio of tears behind the lens that were replaced every hour (i.e., a higher coefficient indicated greater tear exchange). The mean coefficient of tear exchange per hour was 2.5, ranging from 0.2 to 12 (i.e., the entire fluid reservoir was estimated to be replaced on average every 24 min, with a range of 5–300 min). Ko et al. calculated that in order to meet the theoretical oxygen demand of the cornea, a tear exchange coefficient value of 13 was required (entire fluid reservoir replacement every 4.6 min), which was only achieved for one patient [9]. More recently, Paugh et al. [10] used fluorophotometry to quantify tear elimination rates in habitual scleral lens wearers and observed minimal tear elimination during scleral lens wear (0.57 ± 0.6% per minute) compared to soft silicone hydrogel lenses (6.09 ± 2.8% per minute).

Other studies have also examined the tear dynamics of modern high Dk non-fenestrated scleral lenses [7, 8, 12]. After 20–30 min of scleral lens wear, Tan et al. [7] observed a high degree of variability in tear inflow rates; 33% of participants displayed a low rate of tear inflow (>5 min for the dye to enter the fluid reservoir), while 25% displayed a high rate of tear inflow (<30 s). In another study, after 5 h of scleral lens wear, only 10% of participants exhibited a high rate of tear inflow compared with 40% having a low rate [8]. These studies suggest that for approximately one-third of scleral lens wearers, there is minimal tear exchange 20 min after lens application for a non-fenestrated design. The high degree of inter-participant variability observed was also consistent with earlier work by Ko et al. [9]. The effect of modifying the alignment of the scleral lens periphery upon tear exchange has been investigated recently, with a quadrant-specific lens design displaying less tear exchange compared to a spherical landing zone [11].

Improving the current understanding of tear dynamics during scleral lens wear may help to optimise corneal health during scleral lens wear. While modern scleral lenses typically induce minimal central corneal oedema in healthy eyes (~1–3%) [7, 13, 14], oedema can increase in post-surgical corneas (3–18%) [15–17] and is typically greater towards the peripheral cornea [18, 19]. Enhancing tear exchange in such cases may be particularly important if the scleral lens oxygen permeability [20], lens thickness [21] and fluid reservoir thickness [22] have already been optimised to minimise hypoxic stress. Therefore, the primary aim of this pilot study was to develop a Scheimpflug-based image analysis technique to quantify fluid reservoir tear dynamics during short-term fenestrated scleral lens wear.

## Methods

The study was approved by the Queensland University of Technology human research ethics committee and conducted in accordance with the tenets of the Declaration of Helsinki. Informed consent was obtained for each participant following an explanation of the nature of the experiment. The participants and some procedures undertaken in this experiment have been described previously [23, 24].

### Fluid Reservoir Imaging Technique

**Fig. 1:**
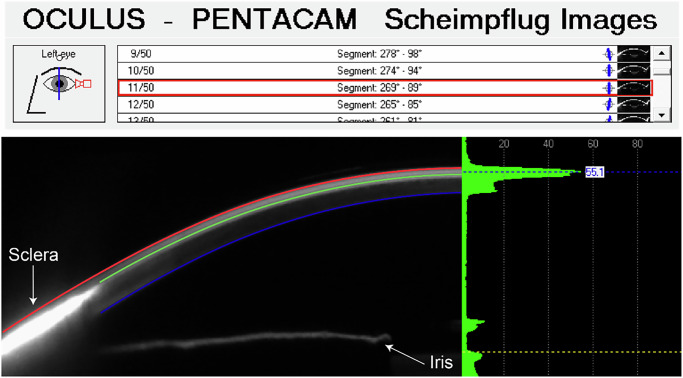
A single cross-sectional Pentacam HR (pentacam.com) Scheimpflug image (left) of a scleral lens on the eye (only visible in the Scheimpflug image with contrast enhancement) with sodium fluorescein within the fluid reservoir, alongside the corresponding densitometry graph (right, scale of 0–100). The red line indicates the posterior scleral lens surface, the green line is the anterior corneal epithelial boundary and the blue line is the corneal endothelial layer. The space between the red and green lines is the fluid reservoir.

The conversion of absorbed blue light to emitted fluorescent yellow light peaks with blue light wavelengths near 490 nm [25], and this coincides closely with the wavelength of light used by the Pentacam HR instrument (Oculus, pentacam.com) (475 nm). The Pentacam uses blue light to capture Scheimpflug images [26, 27] of the anterior segment. However, several studies have also used Scheimpflug imaging to examine the change in the turbidity of the fluid reservoir during scleral lens wear based on densitometry measurements [28–30]. In this study, Scheimpflug images were analysed using customised software to quantify the change in fluid reservoir intensity (fluorescence) during fenestrated scleral lens wear (Fig. 1).

### Participants

Nine adults (mean age ± standard error 30 ± 1 years) completed the experiment. A required sample size of eight was determined using G*Power (psychologie.hhu.de/arbeitsgruppen/allgemeine-psychologie-und-arbeitspsychologie/gpower, version 3.1.9.2) to investigate differences in central corneal oedema, assuming an effect size of 1.17 based on the open eye central corneal oedema data from a corneal rigid lens study (material Dk 100) comparing non-fenestrated and fenestrated lenses (one 0.25 mm fenestration) [31]. The results of this analysis have been reported previously [23]. An initial ophthalmic screening was conducted to exclude participants with any ocular or vision abnormalities, contraindications to contact lens wear, previous ocular injury or surgery or current use of topical medications. Each participant had visual acuity of 0.00 logMAR or better in both eyes. Soft contact lens wearers (*n* = 5) ceased lens wear for 24 h prior to any experimental session, and none of the participants were rigid contact lens wearers.

### Scleral Lens Fitting

The scleral lenses used in this experiment (KATT^TM^, Capricornia Contact Lenses, capcl.com.au) were manufactured in hexafocon B material (Dk 141 × 10^−11^ cm^3^ O_2_(cm)/[(s)(cm^2^)(mmHg)]). They had a 16.5 mm diameter, a spherical landing zone, back vertex power of −1.00 D, back optic zone radius of 7.46 mm, a nominal centre thickness of 300 µm and a single lens fenestration (one 0.3 mm diameter fenestration) located 6.25 mm from the centre of the optic zone, positioned to approximately overlie the limbus. The fenestrated lens used for each participant was selected to achieve an initial central fluid reservoir thickness of ~150 µm to ensure any air bubbles that entered the fluid reservoir remained towards the peripheral cornea [32]. The optimal lens for the left eye of each participant was determined during a trial fitting session using lenses of different sagittal heights. The fluid reservoir thickness was measured using optical coherence tomography (OCT) (Spectralis, Heidelberg, heidelbergengineering.com).

### Measurement Session

Following the screening and initial fitting session, participants attended the laboratory for 90 min of fenestrated scleral lens wear in the left eye only. After application of the optimal fitting lens (as determined at the fitting session) with preservative-free saline (Lens Plus OcuPure, Abbot Medical Optics, abbott.com), a 10 µL drop of 0.091% sodium fluorescein was applied to the bulbar conjunctiva using a 5–40 µL pipette (Labsystems Finnpipette, thermofisher.com). This concentration was selected based on a number of trials to ensure the Pentacam densitometry values remained below 100 (i.e., the fixed upper limit of the Pentacam densitometry scale) to avoid a saturation/ceiling effect. Pentacam measurements were acquired at 0, 5, 10, 15, 20, 30, 60 and 90 min after application of the sodium fluorescein. A 50-line 3D HR (high resolution) scan was captured at each time point while the participant fixated the central internal fixation target of the instrument.

Central and peripheral anterior segment OCT scans were obtained after the first Pentacam measurement and again after 90 min of lens wear prior to lens removal. Central scans were centred on the pupil, and nasal and temporal peripheral scans were obtained while participants maintained fixation on an external target positioned 26.5° laterally. A volumetric scanning protocol was used (3 × ~8 mm horizontal line scans consisting of an average of 20 B-scans, separated vertically by 139 µm).

### Image Processing

**Fig. 2:**
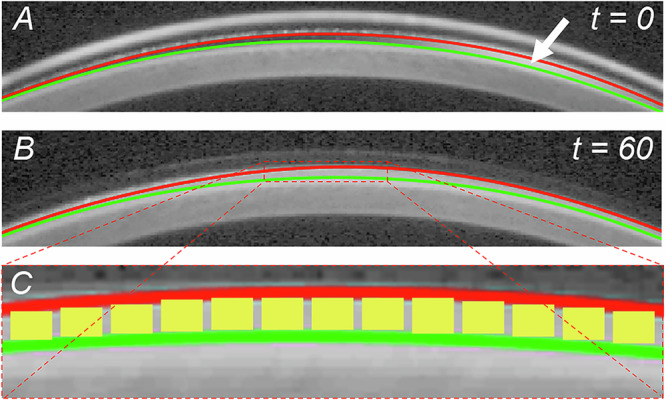
Example of Scheimpflug image segmentation. **A** Image captured immediately after sodium fluorescein application to the conjunctiva, which demonstrates ingress into the fluid reservoir peripherally as indicated by the white arrow (*t* = 0 min). **B** At *t* = 60 min, the central fluid reservoir displays an approximately uniform intensity. The red and green lines are the anterior and posterior fluid reservoir boundaries, respectively. **C** A magnified image of the central fluid reservoir showing the vertical ‘columns’ representing the locations of averaged pixel intensity along the horizontal section of the fluid reservoir.

Four cross-sectional Scheimpflug images along the 0–180°, 45–225°, 90–270° and 135–315° meridians were exported from the Pentacam and analysed by a single examiner using custom-written software. The back surface of the scleral lens and the anterior corneal boundary were annotated in each image to delineate the central fluid reservoir as the region of interest from which to extract the intensity data (Fig. 2). The examiner manually selected nine points along each boundary, which the software then fitted with a spline curve. Care was taken not to include the anterior corneal epithelium or any posterior lens deposits within the region of interest since they have a substantially higher intensity value compared to the fluid reservoir. The intensity of each pixel along the vertical direction between the segmented boundaries of the fluid reservoir was automatically extracted from the image. The intensity value was extracted directly from the grey scale values in the Scheimpflug image, which has a scale ranging from 0 to 255 arbitrary units (AU). This created ‘columns’ of intensity data for each pixel along the horizontal direction, an average intensity metric (average of the four meridians) and a maximum intensity metric (the maximum intensity of the four meridians) was calculated for each ‘column’ for each horizontal pixel location (Fig. 2). However, since there were substantial differences between the maximum and average metric with increasing distance from the image centre, the maximum metric was chosen as the most representative value for data analyses.

OCT images were also exported for analysis using customised software by a single examiner. The software segmented the posterior scleral lens surface, anterior epithelium, anterior stroma and endothelium. Any segmentation errors were manually corrected if required, and the location of the scleral spur was marked in the peripheral scans. Three measurements were averaged for each participant at each time point. Central thickness measurements were constrained to the central 5 mm (0 to 2.5 mm from the corneal apex), and peripheral (−1.0 to 0 mm) corneal thickness measurements were referenced to the location of the scleral spur (0 mm). The measurements were averaged across the nasal and temporal sides. Three OCT line scans from a single image were averaged for each time point.

### Data Analysis

**Fig. 3:**
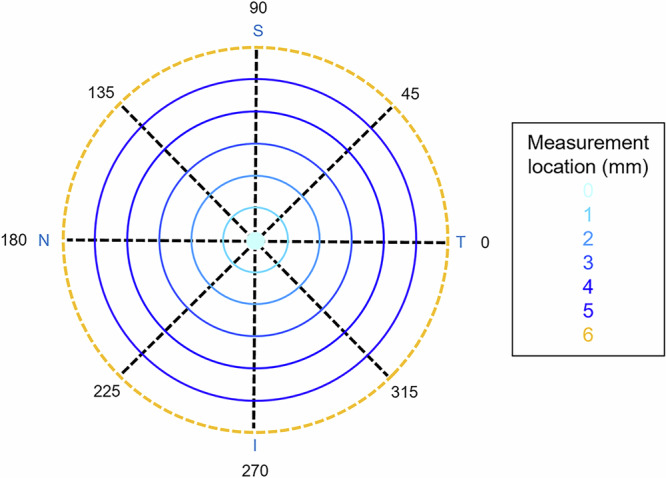
Schematic diagram of data analysis locations. Four Scheimpflug images were extracted for analysis: 0–180°, 45–225°, 90–270° and 135–315° meridians (black dashed lines). The yellow line indicates the approximate location of the scleral lens fenestration (6.25 mm from the lens centre). The maximum and average intensity were extracted at each time point from measurement locations from 0 to 5 mm from the centre of the image along each hemi-meridian.

The four Scheimpflug images selected for analysis at each time point provided data from one central location (four repeated measures) and eight hemi-meridians (one measurement along each hemi-meridian) (Fig. 3). The maximum fluid reservoir intensity was extracted from the centre of each Scheimpflug image (central location, 0 mm) and at 1, 2, 3, 4 and 5 mm locations from the central location along each hemi-meridian. Measurement locations >5 mm from the centre of the image were not considered since for some participants this location displayed a very high intensity value due to reflectivity artefacts from the sclera [33].

### Statistical Analysis

To provide an estimate of the repeatability of the central fluid reservoir intensity measurements, the coefficient of repeatability (CR) was calculated using the four central maximum fluid reservoir intensity measurements extracted from each meridian at each time point during fenestrated lens wear using the formula ${\mathrm{CR}}=1.96\,\times \,\sqrt{2}\,\times \,{\mathrm{SD}}$, where SD is the within-subject standard deviation (i.e., the standard deviation of the four central measurements for each participant at each time point).

Statistical analyses were performed using IBM SPSS (ibm.com), and the normality of the data was confirmed using the Kolmogorov–Smirnov test. A repeated measures analysis of variance (RM-ANOVA) was used to compare the maximum fluid reservoir intensity across the different locations (within-subject effect of location, 0–5 mm from the image centre), at each time point (within-subject effect, 0–90 min) and their interaction, with Bonferroni corrected post-hoc comparisons for significant effects of interest (i.e., the location by time interaction). An additional RM-ANOVA was used to compare the fluid reservoir intensity over time (within-subject effect of time, 0–90 min) for participants characterised as having ‘low’ or ‘high’ flow fluid reservoir dynamics (a between-subject effect). A gradual increase in fluid reservoir intensity during the first 0–20 min of wear, followed by a plateau, was considered ‘low flow’. An increase in fluid reservoir intensity during the first 0–10 min of lens wear, followed by a decrease from the peak intensity, which then plateaued, was considered ‘high flow’. The Greenhouse-Geisser correction was used to adjust for lack of sphericity, when required, based on Mauchly’s test. A two-tailed, unpaired *t*-test was used to compare the magnitude of central and peripheral stromal corneal oedema between the two tear dynamics groups (‘low’ and ‘high’ flow). Data are presented as the mean ± standard error, and *p* values < 0.05 were considered statistically significant.

## Results

### Repeatability of Central Fluid Reservoir Intensity Over Time

**Fig. 4:**
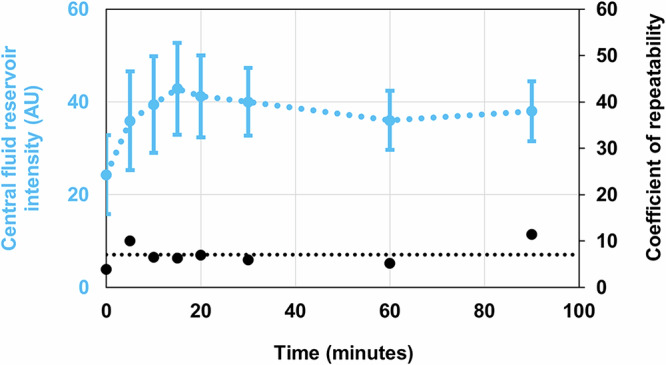
The mean ± standard error maximum central fluid reservoir intensity (blue, arbitrary units, AU) within the fluid reservoir during 90 min of fenestrated scleral lens wear (blue, left axis). Black circles represent the group mean coefficient of repeatability (four central measurements) for each measurement time point, and the black line indicates the mean coefficient of repeatability across all measurement time points (black, right axis).

The average CR of the central (0 mm location) fluid reservoir intensity ranged from 4 to 11 AU (on a scale of 0–255 AU) across all measurement time points (Fig. 4). The average CR across all time points was 7 AU.

### Variation in Central Fluid Reservoir Intensity Over Time

There was no significant variation in central fluid reservoir (0 mm location) intensity over time (*F* = 3.09, *p* = 0.07) when averaged across all measurement locations. Figure 4 displays the slight increase in intensity during the first 15 min of lens wear (mean increase of 18 AU), which then decreased slightly for the remainder of lens wear.

### Variation in Fluid Reservoir Intensity with Measurement Location

Fluid reservoir intensity varied with measurement location (*F* = 85.57, *p* < 0.001). Averaged across all time points, post-hoc pairwise comparisons revealed that the fluid reservoir intensity at the 4 mm (54 ± 9 AU) and 5 mm (100 ± 14 AU) locations was greater than the central 0 mm location (37 ± 7 AU) (*p* = 0.047 and *p* = 0.04, respectively).

### Variation in Fluid Reservoir Intensity with Measurement Location Over Time

**Fig. 5:**
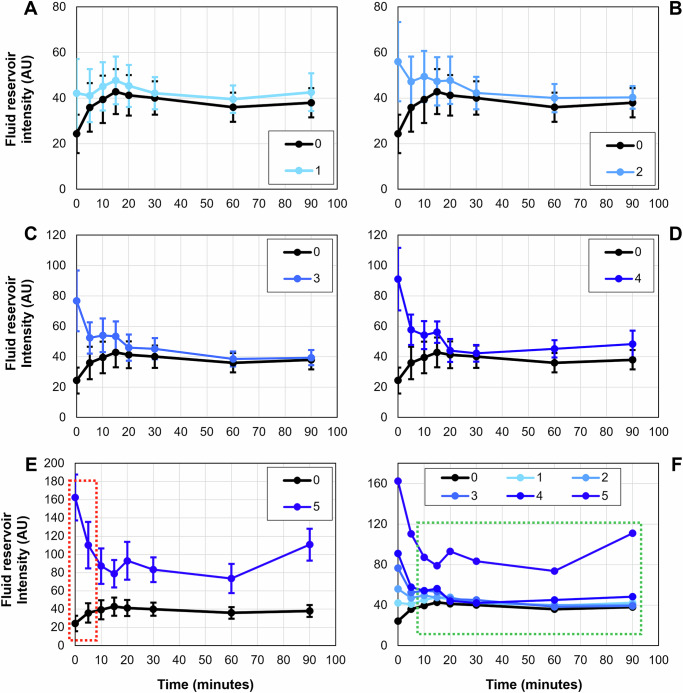
The mean ± standard error fluid reservoir fluorescein intensity (arbitrary units, AU) over the 90 min of fenestrated lens wear for the different locations relative to the central 0 mm measurement (**A**: 1 mm, **B**: 2 mm, **C**: 3 mm, **D**: 4 mm and **E**: 5 mm). Plot **F** displays the mean at each location (0–5 mm) without error bars for ease of comparison. Note that the *y*-axis differs across plots **A**–**F**. Data points within the dashed red line (**E**) are significantly different between the 0 and 5 mm locations for each time point. Data points within the dashed green line (**F**) are not statistically different at each time point.

There was a significant interaction between measurement location and time (*F* = 2.46, *p* = 0.04). Figure 5 displays the change in fluid reservoir intensity over time for each non-central location compared to the central 0 mm location. RM-ANOVA revealed that the fluid reservoir intensity was significantly greater at the 5 mm location compared to the central location immediately after sodium fluorescein application (138 ± 25 AU greater at 5 mm, *p* < 0.001) and again after 5 min (75 ± 17 AU greater at 5 mm, *p* = 0.04) (Fig. 5E). However, after 10 min this difference had reduced to 48 ± 16 AU (*p* = 0.40), suggesting that the central and peripheral fluid reservoir had reached a similar intensity 10 min after fluorescein application.

### Inter-subject Variability and Corneal Oedema

**Fig. 6:**
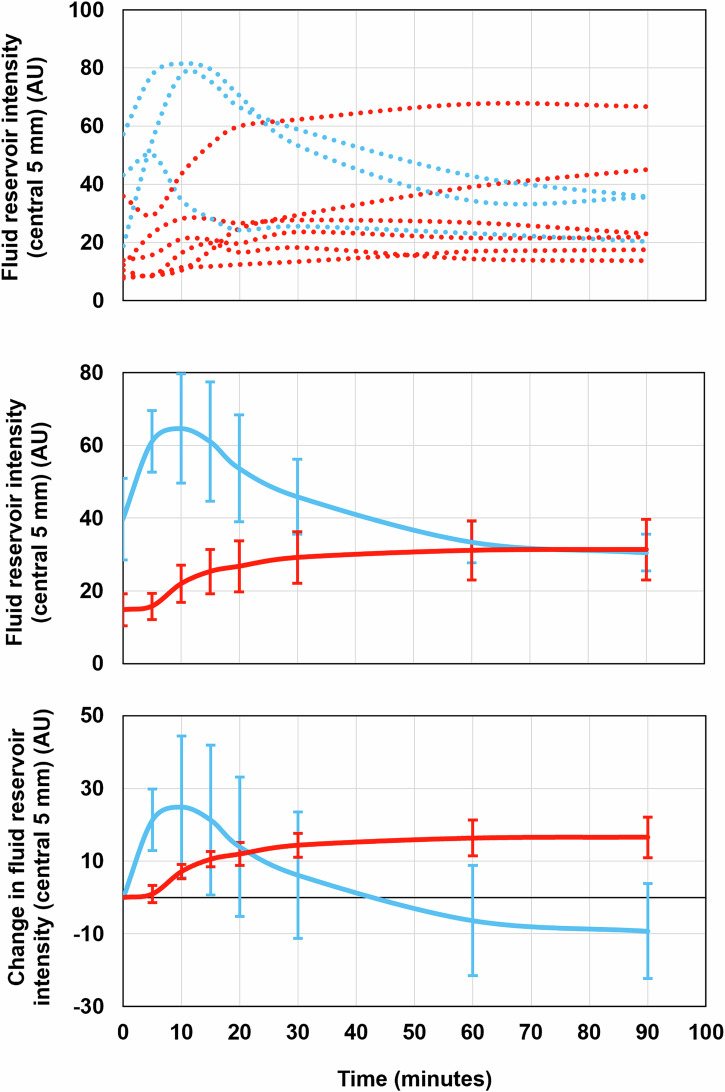
Top panel: the mean fluid reservoir intensity data (across the central 5 mm) for each participant demonstrating either ‘high flow’ (blue lines, *n* = 3) or ‘low flow’ (red lines, *n* = 6). Middle panel: The mean fluid reservoir intensity data (across the central 5 mm) for each group. Lower panel: The relative mean ± standard error change in fluid reservoir intensity data (across the central 5 mm) for each group (relative to the baseline measurement at 0 min). Error bars are the standard error. AU arbitrary units.

Figure 6 highlights the two patterns of change in fluid reservoir intensity (averaged across the central 5 mm), observed over 90 min of lens wear. The most common pattern (‘low flow’, red lines in Fig. 6) was a gradual increase in fluid reservoir intensity during the first 0–20 min of wear, followed by a plateau (6 of 9 participants). Three participants displayed an increase in fluid reservoir intensity during the first 0–10 min of lens wear followed by a decrease from the peak intensity which then plateaued (‘high flow’, blue lines in Fig. 6). RM-ANOVA revealed a greater increase in fluid reservoir intensity for the ‘high flow’ group compared to the ‘low flow’ group from 0 to 15 min after lens application (all *p* < 0.05). The magnitude of stromal corneal oedema after 90 min of fenestrated lens wear was slightly greater for the ‘low flow’ group (*n* = 6, mean central oedema 0.93 ± 0.63%, peripheral oedema 0.63 ± 1.18%) compared to the ‘high flow’ group (*n* = 3, mean central oedema 0.25 ± 0.17%, peripheral oedema 0.28 ± 1.91%) but this difference did not reach statistical significance (central *p* = 0.39, peripheral *p* = 0.87).

## Discussion

This pilot study examined the change in the fluorescent intensity of the fluid reservoir during short-term fenestrated scleral lens wear using Scheimpflug imaging and customised image analysis software. The developed technique displayed a CR of 7 AU for the central fluid reservoir; however, the region of analysis requires careful selection to remove potential image artefacts that can increase intensity readings significantly, such as the anterior epithelium, the sclera and deposits on the posterior lens surface. While this study only considered the tear dynamics of fenestrated scleral lenses with sodium fluorescein applied to the conjunctiva (the ‘out-in’ method), the imaging technique could also be used to track tear flow when a dye is applied within the scleral bowl prior to application (the ‘in-out’ method).

A major finding of this pilot study was that the ingress of sodium fluorescein into the fluid reservoir and the subsequent mixing of the dye with the preservative-free saline within the reservoir, equalised between central and peripheral locations relatively quickly for a scleral lens with a single 0.3 mm diameter fenestration fitted with an initial central fluid reservoir thickness of ~150 µm. Figure 5 highlights that the fluid reservoir fluorescent intensity across the central 10 mm (0–5 mm locations) was stable after 10 min of lens wear. Only Ko et al. [9] have previously examined fluid reservoir fluorescence during 1 h of scleral lens wear with a 1–1.5 mm diameter limbal fenestration. In contrast to the current experiment, they mixed fluorescein with saline in the scleral lens bowl prior to lens application and examined the dilution of the fluid reservoir with the ingress of natural tears (the ‘out-in’ method). Ko et al. [9] observed a high degree of variability in tear exchange between participants and the fellow eyes of individuals, similar to the findings of the current study (Fig. 6, top panel).

Two distinct patterns of fluid reservoir dynamics were observed in the current experiment (Fig. 6); (1) ‘low flow’ characterised by a gradual increase in fluorescent intensity over 30 min followed by a plateau, or less frequently (2) ‘high flow’ characterised by a faster and more prominent increase in fluorescent intensity over 10 min followed by an equally rapid decrease (dispersion of fluorescein or replacement with the natural tears) and then a plateau. For participants with a ‘low flow’ pattern, it is likely that there was minimal tear exchange via the fenestration or regions of misalignment between the landing zone and conjunctiva. In contrast, the fluid reservoir dynamics of the three participants who displayed a ‘high flow’ pattern suggests that sodium fluorescein entered the reservoir, mixed with the preservative-free saline and then reduced in intensity due to further tear exchange either through the fenestration or as a result of landing zone misalignment. Future studies should include a non-fenestrated scleral lens control condition using the same lens design without a peripheral fenestration to determine if the increase in tear exchange occurs via the fenestration or beneath the landing zone, perhaps due to reduced suction forces.

While previous work has shown that scleral lenses with a single peripheral fenestration induce slightly less central oedema [21] and substantially less peripheral oedema [22] compared to non-fenestrated lenses, this study explored the level of oedema associated with two different patterns of fluid reservoir dynamics (Fig. 6). The six participants who exhibited less tear exchange during scleral lens wear exhibited 3.72× more central and 2.25× more peripheral corneal oedema compared to the three participants with a rapid ingress of tears. While this difference was not statistically significant, the association between fluid reservoir dynamics and scleral lens-induced oedema should be investigated in a larger population. In a sample of four eyes fitted with fenestrated scleral lenses (a single limbal fenestration), Ko et al. [9] concluded that only one of the four eyes tested was receiving an adequate oxygen supply to meet the theoretical corneal metabolic demand, suggesting that tear dynamics may vary substantially with each lens fit.

This exploratory study was limited by its relatively small sample size (which was selected to examine variations in corneal oedema reported previously [23, 24]), particularly since there is substantial variation in fluid reservoir dynamics between individuals, based on the results of the current study and previous investigations [7–9]. The current study was also restricted to a single lens design using only one 0.3 mm diameter peripheral fenestration. Future studies utilising different scleral lens designs with a greater number of fenestrations of varying diameter (including a non-fenestrated lens control condition) may reveal different patterns of fluid reservoir dynamics. A disadvantage of the Scheimpflug imaging approach is that the fluid reservoir towards the periphery can be affected by artefacts arising from reflections off the sclera, which can limit the amount of usable peripheral data. Another reflectometry method has recently been developed to avoid this artefact and quantify tear exchange over a much larger area during scleral lens wear [34]. However, the reflectometry approach cannot differentiate between fluorescent changes anterior to the scleral lens surface (pre versus post lens fluorescence), while the Pentacam method can isolate analysis to the fluid reservoir due to the ability to select the region of interest within the cross-sectional Scheimpflug images.

In conclusion, a method was developed using Scheimpflug imaging and customised software to quantify changes in fluid reservoir fluorescent intensity during fenestrated scleral lens wear due to the ingress of externally applied sodium fluorescein (the ‘out-in’ method). The intensity of sodium fluorescein within the fluid reservoir equalised between central and peripheral locations within 10 min for the fenestrated lens design used in this experiment. Two patterns of fluid reservoir fluorescent intensity dynamics were observed: a gradual increase and plateau (‘low flow’), or a rapid increase followed by a decrease and plateau (‘high flow’). Participants exhibiting ‘high flow’ also displayed less central and peripheral corneal oedema, but this difference was not statistically significant. Future research utilising the developed imaging and analysis technique is required to provide further insights into tear exchange during scleral lens wear with different fenestration sizes and configurations, and their potential impact upon corneal oedema in a larger sample size.

## Data Availability

All data supporting the findings of this study are available within the paper.
